# Chronotropic Response and Risk Factors for Cardiovascular Disease in Patients with Rheumatoid Arthritis: A Cross-Sectional Study

**DOI:** 10.3390/jcm12237256

**Published:** 2023-11-23

**Authors:** Ahmad M. Osailan, George S. Metsios, Joan L. Duda, George D. Kitas, Jet J. C. S. Veldhuijzen van Zanten, Ragab K. Elnaggar

**Affiliations:** 1 Health and Rehabilitation Sciences Department, College of Applied Medical Sciences, Prince Sattam bin Abdulaziz University, Alkharj 16278, Saudi Arabia; 2 Department of Rheumatology, Dudley Group NHS Foundation Trust, Dudley DY1 2HQ, UKgeorge.kitas@nhs.net (G.D.K.);; 3 School of Sport, Exercise and Rehabilitation Sciences, University of Birmingham, Birmingham B15 2TT, UK; j.l.duda@bham.ac.uk; 4 School of Physical Education and Sport Science, University of Thessaly, 42130 Trikala, Greece; 5 Department of Physical Therapy for Pediatrics, Faculty of Physical Therapy, Cairo University, Giza 12613, Egypt

**Keywords:** exercise testing, chronotropic response, cardiovascular diseases, rheumatoid arthritis

## Abstract

Background: Individuals with rheumatoid arthritis (RA) are at a high risk of cardiovascular diseases (CVD). A reduced chronotropic response (CR), which produces exercise intolerance, is known to be a contributing factor to CVD and mortality. Studies have shown that patients with RA have a reduced CR. However, knowledge of CR-related factors in patients with RA is limited. This study aimed to explore CR-related factors, including CVD risk factors, inflammatory markers, and cardiorespiratory fitness (VO_2PEAK_). Methods: A total of 106 RA patients underwent a treadmill test, heart rate monitoring, and various assessments, including serological CVD risk factors, inflammatory markers, and VO_2PEAK_. Results: A total of 34% of participants demonstrated a reduced CR (≤80%). Body mass index, HOMA, hsCRP, and fibrinogen were inversely related to CR, while HDL, QUICKi, VO_2PEAK_, and RER exhibited a positive association. HDL and VO_2PEAK_ emerged as independent CR-related factors in regression analysis. Conclusions: The current findings suggest that reduced CR in RA is associated with several CVD risk factors, inflammatory markers, and cardiorespiratory fitness. Future studies should investigate the effects of controlling these associated variables on CR in patients with RA.

## 1. Introduction

Cardiovascular diseases (CVD) have emerged as a major cause of more than 40% of mortality in patients with rheumatoid arthritis (RA) [1]. The risk of cardiovascular disease (CVD) is mediated by an array of both traditional and non-traditional factors. Traditional risk factors include hypertension, dyslipidemia, and physical inactivity. Non-traditional risk factors, such as systemic inflammation, autonomic nervous system (ANS) dysfunction, hyperuricemia, and genetic predispositions, have also been identified as significant contributors to CVD risk [2,3]. ANS plays a major role in regulating the cardiovascular system during exercise, including heart rate (HR) response to stress and physical activity [4].

The manifestation of CVD symptoms in patients with RA can vary based on numerous factors, such as the severity and activity of RA, the presence of other risk factors for CVD (e.g., hypertension, diabetes, smoking), management of RA, and disease activity score [5,6]. People with RA are estimated to have a 50% higher risk of experiencing a cardiovascular event, such as a heart attack or stroke, compared to those without RA [7,8].

The chronotropic response (CR), a fundamental aspect of cardiovascular functionality, plays a pivotal role in determining heart health. CR is the heart’s inherent ability to modulate its rate in response to various levels of physical activity or emotional stress. This response is critical for maintaining optimal blood flow and oxygen supply throughout the body during different physiological states. In patients with rheumatoid arthritis (RA), however, this adaptive mechanism is often significantly impaired [9]. RA has been increasingly recognized for its systemic effects, including its impact on cardiovascular health. The compromised CR in RA patients leads to an inability to adequately increase HR during exercise or stress, resulting in exercise intolerance. This intolerance not only limits physical capacity but also exacerbates overall cardiovascular risks [10]. In individuals with RA, impaired CR is linked with a higher incidence of ischemic heart disease, heart failure, and other cardiovascular complications, underscoring the need for comprehensive cardiovascular assessment and management in this population.

An abnormal CR, also known as chronotropic incompetence, refers to the inability of the heart to adjust its rate appropriately in response to varying levels of activity or stress, such as during exercise [11]. Normally, the HR should increase proportionally to the intensity of physical activity or stress. Several factors contribute to abnormal CR, such as age, cardiac autonomic neuropathy, and the use of certain medications. Age-related changes in the ANS and the sinoatrial node are linked to a decrease in the maximum heart rate and chronotropic incompetence [12]. Cardiac autonomic neuropathy, common in conditions like diabetes, can impair heart rate variability and lead to a blunted heart rate response to exercise [13]. Medications, particularly beta-blockers and certain calcium channel blockers, can influence heart rate responses by modulating the activity of the ANS and cardiac pacemaker cells [11].

RA is a systemic inflammatory autoimmune disease that can affect various organs, including the heart. The systemic inflammation present in RA patients could influence the autonomic regulation of the heart rate, potentially leading to chronotropic incompetence [14]. Moreover, people with RA often have co-existing conditions such as diabetes mellitus and are also frequently on medications like non-steroidal anti-inflammatory drugs (NSAIDs) and corticosteroids, which can affect heart rate responses [15,16]. Additionally, the physical inactivity that might accompany RA due to joint pain and stiffness could further contribute to an altered CR by affecting overall cardiovascular fitness and autonomic tone [17]. Further, it was also suggested that the alterations in heart rate response may suggest a potential link between RA and cardiovascular ANS dysfunction. This mechanism was investigated in a study by Roubille et al. [18], which proposed that chronic inflammation associated with RA may lead to autonomic dysfunction, which, in turn, affects the heart’s ability to respond appropriately to changes in physiological demands. Understanding and monitoring CR in RA patients can be crucial in evaluating their CVD risk and tailoring their therapeutic regimens to minimize potential cardiac complications. Therefore, these findings highlight the need for further research to elucidate the underlying mechanisms and potential cardiovascular implications in patients with rheumatoid arthritis.

Clinically, graded exercise testing (GXT) is considered a method for assessing the risks and prognoses of people with subclinical CVD. GXT and the assessment of cardiorespiratory fitness (CRF) hold substantial importance in managing RA due to their role in evaluating and enhancing cardiovascular and overall physical health [19]. GXT assists in understanding cardiovascular function and exercise tolerance, which is crucial given the elevated risk of CVD in RA patients. ANS regulates the HR at rest and during GXT. GXT is associated with increased sympathetic and decreased parasympathetic activity, reflected in an increased HR response commonly defined as a CR [20]. Abnormal CR during GXT may indicate ANS dysfunction, which has been recognized as a strong and independent predictor of all-cause mortality [21].

There is a lack of data regarding CR and its associated factors in patients with RA. Peçanha et al. [9] showed that patients with RA (n = 27) had reduced CR and slower post-exercise heart rate recovery than healthy individuals (n = 14). However, no investigation was made to evaluate the factors associated with abnormal CR. Identifying the multitude of CVD risk factors that influence abnormal CR levels in individuals with RA is crucial. Recognition and understanding of these factors are essential for mitigating the CVD risk in the RA population. Thus, the purpose of the current study was to evaluate CR in patients with RA and investigate the factors associated with CR. Reduced CR has been hypothesized to be associated with CVD risk factors including cardiorespiratory fitness and markers of inflammation.

## 2. Methods

### 2.1. Study Population

One hundred and six individuals diagnosed with rheumatoid arthritis (RA) met the revised criteria set by the American College of Rheumatology [22]. The data utilized in this study were gathered from RA patients who were recruited from the outpatient clinics of the Dudley Group NHS Foundation Trust, UK, between October 2011 and 2014. These patients participated in an exercise intervention study, registered under the trial number ISRCTN04121489. Participants were excluded if they had recent joint surgery within the last six months, atrial fibrillation, fibromyalgia, established cardiovascular disease (CVD), or any comorbidities that made exercise inadvisable, according to the American College of Sports Medicine (ACSM) [23]. Every participant gave informed consent for their involvement in the study. The research adhered to the ethical guidelines stipulated by the Declaration of Helsinki and received approval from the National Research Ethics Committee (1-/H1206/59, approved on 3 February 2011).

### 2.2. Study Protocol

A comparable protocol has been previously described by Osailan et al. [2]. Participants were required to visit the research laboratory on two separate occasions. At the first visit, blood samples were taken from fasting participants. On the second visit, various measurements were conducted. Participants’ brachial blood pressure was taken while they were seated, using an electronic sphygmomanometer (Datascope Accutor, Mahwah, NJ, USA). Their height was measured to the nearest 0.5 cm using a standard height measure (Seca 214 Road Rod), and their weight and body mass index (BMI) were assessed using a Tanita BC 418 MA Segmental Body Composition Analyzer (Tanita Corporation, Tokyo, Japan). During this same visit, participants were equipped with suitably sized masks covering their nose and mouth for the purpose of conducting inspired and expired gas analyses. Heart rate was monitored using a 12-lead electrocardiogram (ECG) (12-channel ECG Custo Cardio 200, Custo Med, Leipzig, Germany). A two-minute baseline measurement was taken while participants were seated to assess resting heart rate and oxygen consumption volumes. Subsequently, a graded exercise test (GXT) was performed, followed by a six-minute recovery period post-GXT.

### 2.3. Graded Exercise Test (GXT) Protocol

The graded exercise test (GXT) was conducted on a treadmill (HP Cosmo Mercury; Nussdoerf-Traunstien, Germany), and it was customized based on each participant’s fitness level and physical capabilities [24]. The initial two minutes of the test began at a speed chosen by the participant (roughly 3.5–4 kph) with no incline, and then the speed was gradually increased up to the participant’s maximum brisk walking pace. Starting from the third minute, the GXT proceeded with the walking speed attained in the initial two minutes, but with a 1% incline. The incline was then raised by 1% at the end of each subsequent minute. Breath-by-breath analysis of inspired and expired gases was performed using a Metalyzer 3B (Cortex, Leipzig, Germany), which helped in determining the peak volume of oxygen uptake (VO_2PEAK_). Electrocardiograms (ECG) were continuously recorded during the exercise and throughout the recovery phase. The test was concluded when the participant either voluntarily stopped due to exhaustion, was unable to continue the test, or if any relative or absolute contraindication based on the American ACSM criteria was identified [23]. Following the termination of the test, participants’ blood pressure and HR were monitored for an additional five minutes.

### 2.4. Outcome Measures

#### 2.4.1. Chronotropic Response (CR)

CR was determined as the percentage calculated by subtracting the resting heart rate (HR) from the achieved peak HR, and then dividing the result by the difference between the age-predicted maximum HR and the resting HR [25]. The HR readings were obtained from a 12-lead ECG using a 12-channel ECG Custo Cardio 200 (Custo Med, Leipzig, Germany).

#### 2.4.2. Cardiorespiratory Fitness (VO_2PEAK_)

Peak aerobic capacity was assessed during GXT using a calibrated breath-by-breath gas analyzer. The measurements of inspired and expired gases were averaged every 2 s. Oxygen volume (VO_2PEAK_) readings were refined by computing the average VO_2PEAK_ every 28 s, which involved averaging 14 consecutive VO_2PEAK_readings (mL/min). Using 28 s intervals can capture meaningful changes in oxygen consumption without too much fluctuation [26]. VO_2PEAK_ was defined as the highest VO_2PEAK_ during the test and was expressed as mL/min/kg.

#### 2.4.3. Blood Sample Analysis

Fasted blood samples were analyzed for serological risk factors for CVD, including total cholesterol, high-density lipoprotein (HDL), low-density lipoprotein (LDL), and triglycerides [27,28]. Insulin resistance was assessed using the homeostasis model assessment (HOMA) [29] and insulin sensitivity was evaluated using the quantitative insulin sensitivity check index (QUICKi) [30]. Inflammatory markers, including erythrocyte sedimentation rate (ESR), which measures the rate at which red blood cells sediment in a period of one hour [31], high-sensitivity C-reactive protein (hsCRP) [32], fibrinogen using the Clauss method [33], and white blood cells (WBC), were assessed. Analyses were performed using routine laboratory procedures in the hospital’s laboratory.

### 2.5. Statistical Analysis

Statistical analyses were performed using SPSS29 (Chicago, IL, USA). The normality of the data was assessed using the Kolmogorov–Smirnov test. Normally distributed variables are presented as means, standard deviations, and medians and interquartile ranges for non-normally distributed variables. Bivariate correlation using Spearman’s correlation analysis was used to assess the relationship between the CR and the study’s primary outcome variables. Linear regression was used using the enter method, with CR as the dependent variable, and all variables significantly associated with CR in the correlation analysis were used as independent variables. The level of significance was set at *p* ≤ 0.05.

## 3. Results

The demographic characteristics and primary outcome variables of the 106 RA patients are presented in Table 1. The most common comorbidities are presented in Table 1.

### 3.1. Correlation Analysis

**Figure 1:**
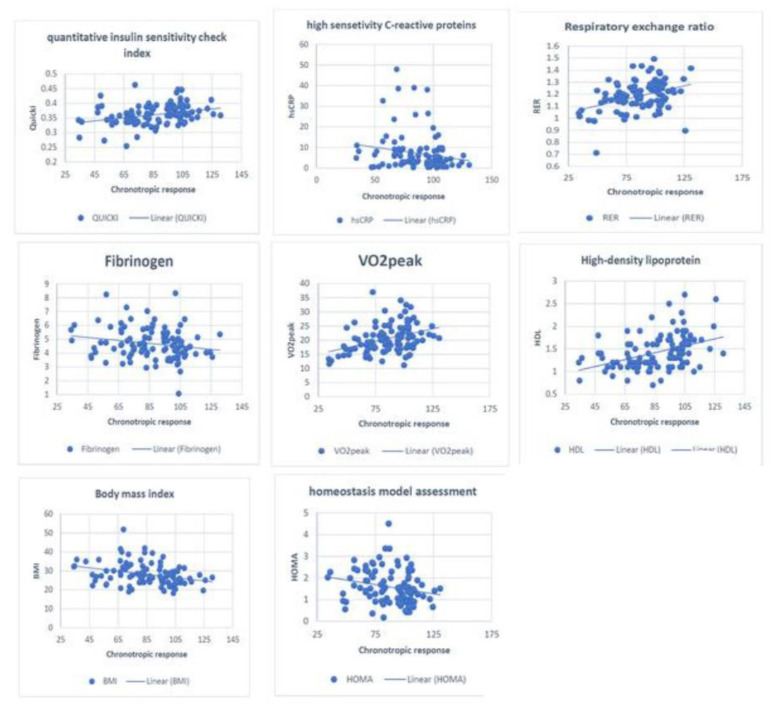
Correlation between CR and multiple CVD risk factors.

Correlation analysis showed a significant inverse relationship between CR and BMI (*r* = −0.26, *p* = 0.01) (Figure 1), HOMA (*r* = −0.26, *p* = 0.01), hsCRP (*r* = −0.2, *p* = 0.05), and fibrinogen (*r* = −0.2, *p* = 0.05), whereas CR was significantly correlated with VO_2PEAK_ (*r* = 0.34, *p* < 0.001) (Figure 1), RER (*r* = 0.33, *p* = 0.001) (Figure 1), HDL (*r* = 0.38, *p* < 0.001) (Figure 1), and QUICKI (*r* = 0.22, *p* = 0.04) (Figure 1) (Table 2).

### 3.2. Linear Regression

Linear regression was used to identify the factors associated with CR (dependent variable), including all variables (independent variables) that were significantly correlated with CR. A model that included BMI, VO_2PEAK_, RER, HDL, HOMA, QUICKi, hsCRP, and fibrinogen explained 37% of the variation in CR [F(8,88) = 6.3, *p* < 0.001, R^2^ = 0.37]. RER and HDL levels were the only two variables independently associated with CR (Table 3). The model was tested for collinearity, and no influence was found.

## 4. Discussion

The current study explored CR in patients with RA, conventional CVD risk factors, and inflammatory markers associated with CR. The study showed that more than one-third of the sample had reduced CR [34] based on the cut of value cut-off value (≤80%) [34]. Several factors showed a weakly significant inverse association with CR, including BMI, HOMA, and inflammatory markers (hsCRP and fibrinogen). Additionally, several variables including VO_2PEAK_, RER, HDL, and QUICKI were moderately and significantly associated with CR. When all the associated factors were entered into a linear regression, a model explained more than one-third of the variation in CR and showed that RER and HDL were independently associated with CR. These results indicate that several CVD risk factors and inflammatory markers contribute to changes in CR, and cardiorespiratory fitness and HDL level are two independent factors that contribute to changes in CR.

CR is the physiological adaptation of HR to the required activity level [35]. This physiological adaptation has been reported to be a strong and independent predictor of morbidity and mortality [36]. Although the use of CR is limited due to the lack of consensus regarding the cut-off value for reduced CR, most studies reported failure to achieve ≤ 80% during graded exercise testing [11]. In this study, the most frequently reported cut-off value was utilized, although alternative cut-off values, such as 85% or 70%, are also used [37]. A reduced CR may reflect an imbalance between the two arms of the ANS (sympathetic and parasympathetic nervous system), which regulates HR among other physiological functions. For example, if the parasympathetic nervous system is overly dominant or the sympathetic nervous system is insufficiently active, it could lead to a blunted CR [38]. The heart may not accelerate as it should during exercise, limiting exercise capacity. This imbalance may compromise exercise performance and could have broader implications for CVD. In this context, given the potential influence of chronic inflammation in RA, which may disrupt the balance between the sympathetic and parasympathetic nervous systems, this may increase the risk of CVD mortality in this population [39].

To the best of our knowledge, this is the first study to report factors associated with CR in patients with RA. A previous study assessed CR and heart rate recovery in (n = 27) females with RA in comparison to (n = 14) age- and BMI-matched controls [9]. The study reported that people with RA had reduced CR and slower post-exercise heart rate recovery, indicating reduced cardiac autonomic functioning in this population. Our results add to these findings since no investigation of factors associated with CR was reported in a previous study. The current study highlights the association between CR and multiple risk factors for CVD in patients with RA.

Few studies have investigated the factors associated with CR in clinical populations. In the Framingham Heart Study, smoking was associated with reduced CR [40]. An association between cardiorespiratory fitness and CR has been reported in individuals with compensated heart failure [41]. Franssen et al. [42] reported an inverse association between systemic inflammation and CR in adolescents with obesity. A previous study found a significant association between reduced CR and the risk of carotid atherosclerosis among healthy people [43]. Interestingly, the latter study reported a significant association between multiple conventional CVD risk factors and carotid atherosclerosis. Only cardiorespiratory fitness was associated with CR among individuals with heart failure, whereas BMI was not significantly associated with CR in this population [44]. Although all previous studies examined some of the factors related to CR in a different population, the current study reported gathered multiple conventional CVD factors as well as disease-related factors (e.g., inflammatory markers) in patients with RA. Due to the variation in methodologies and cohorts, comparing and contrasting the current study’s findings with those of previous studies is difficult. Nevertheless, the current study confirmed some of the earlier findings in underdiagnosed populations at a high risk of developing CVD [7].

Our current findings suggest that many factors are related to reduced CR in patients with RA. Higher BMI, insulin resistance (HOMA), and inflammation (hsCRP and fibrinogen) were related to reduced CR. Furthermore, better CR was related to good cardiorespiratory fitness (VO_2PEAK_ and RER), HDL, and better insulin sensitivity (QUICKi). This may not be entirely surprising, considering that most of the above factors are risk factors for CVD and that CR was found to be predictive of all-cause mortality among healthy people [45] and adverse CVD outcomes among people with diabetes [10]. This may indicate that a blunted HR response to exercise in people with RA may be associated with uncontrolled or undermanaged CVD risk factors, which subsequently increases the risk of CVD.

With regards to the association between CR and HDL, a cautious interpretation of the association must be exercised considering the lipid paradox in people with RA. The functionality of lipids, particularly HDL, may be compromised in RA. This is due to the anti-inflammatory and atheroprotective roles of lipids like HDL might be impaired [46].

Reduced CR in individuals with RA can be attributed to a blunted HR response (blunted sympathetic nervous activity and parasympathetic withdrawal) during exercise. This could be explained by the desensitization of β-adrenergic receptors, causing heightened sympathetic activity similar to those experienced by people with heart failure [47]. This excessively heightened sympathetic activity can lead to β-agonist-stimulated muscle contractility [44]. However, this hypothesis requires further investigation in patients with RA.

### Clinical Implication

The observed correlation between reduced cardiorespiratory fitness and elevated inflammatory markers in RA patients underscores the multifaceted impact of chronic inflammation on cardiovascular health. The findings of the current study not only reinforce the importance of regular cardiovascular monitoring in RA patients but also highlights the potential role of targeted anti-inflammatory therapies in improving cardiorespiratory fitness. Furthermore, the inverse relationship between CR and markers like BMI and HOMA indicates a potential avenue for intervention through lifestyle modifications. Tailored exercise programs, designed with consideration for the unique challenges faced by RA patients, could prove instrumental in enhancing cardiorespiratory fitness, thereby mitigating the elevated cardiovascular risk associated with RA. Future studies should explore the longitudinal effects of such interventions on cardiorespiratory fitness and overall cardiovascular health in this population.

CR holds significant clinical importance as it reflects the function of the ANS. However, it is not utilized in clinical settings. Hence, incorporating CR measurements during routine GXT is essential. Assessment of CR may help in risk stratification and modification of management strategies to avoid the worsening of factors that may eventually lead to CVD in patients with RA. For example, since better cardiorespiratory fitness was associated with better CR, early intervention with exercise training programs in people with reduced CR may help improve CR by improving baroreflex sensitivity and reducing the risk of CVD. Furthermore, the positive relationship between HDL and CR can be explained by the anti-atherosclerotic effect of HDL in stimulating cholesterol efflux [48], which may prevent endothelial damage that can result from cholesterol. This endothelial damage could increase arterial stiffness, subsequently influencing HR response during exercise [49]. However, the exact role of HDL in people with RA remains to be investigated in detail. Nevertheless, CR should be addressed to prevent future CVD in patients with RA as it is related to a poor cardiometabolic profile.

This study has several limitations. Causality between the variables should not be determined because of the study’s cross-sectional design. However, to our knowledge, this is the first study to explore the relationship between CR and multiple CVD factors in patients with RA. In the current study, no comparison group was used, similar to Peçanha et al. [9], as our study aimed to explore factors associated with CR in patients with RA. The lack of information about the medication used is also another limitation. This lack of pharmacological data hampers our ability to comprehensively evaluate the influence of these medications on CR response in the RA population. Future longitudinal studies are warranted to investigate the prognoses of these factors and whether they can lead to CVD and CVD mortality in patients with RA.

In conclusion, the results of the current study showed that an attenuated HR response to exercise measured by CR was associated with multiple risk factors. Most associated factors are determined to have a predictive value for future CVD. Thus, the control and management of these factors should be implemented to prevent and minimize future CVD in this population. Additionally, this needs to be investigated if, for example, improving cardiorespiratory fitness positively influences CR and may protect against future CVD in RA. Furthermore, the current study may also add to the importance of utilizing CR measurement of patient with RA during routine cardiorespiratory fitness assessment.

## Figures and Tables

**Table 1:** Demographic characteristics and main outcome variables.

Characteristics	Value
Age (years)	54.5 ± 12.3
Gender (%)	68.9% ♀
Weight (kg)	76 (64.1–89.9)
Height (m)	1.66 (1.59–1.74)
BMI (kg/m^2^)	27.3 (24–30.9)
RA diseases duration (years)	7.6 ± 8.8
Comorbidities	
Diabetes	6.8%
Hypertension	33%
Smoking	45.6% ex-smoker, 9.7% smoker
Osteoarthritis	27.2%
Cardiorespiratory measurements	
Reduced CR (≤80%) %	34%
Resting SBP (mmHg)	134 ± 16.7
Resting DBP (mmHg)	81 ± 9.9
Resting HR (bpm)	80 ± 12.5
Peak HR (bpm)	155 (139–169)
VO_2PEAK_ (ml/kg/min)	20.6 ± 5
VO_2PEAK_ (L/min)	1.6 (1.3–1.8)
Peak RER	1.2 ± 0.12
Chronotropic response	87.8 (72.2–103.4)
Serological measurements	
LDL (mmol/L)	3.1 (2.5–3.6)
HDL (mmol/L)	1.4 (1.1–1.65)
Triglycerides (mmol/L)	1.1 (0.8–1.4)
WBC (10^9^/L)	6.8 ± 2.3
HOMA	1.5 (0.9–2.1)
QUICKi	0.4 ± 0.03
hsCRP (mg/L)	4.26 (1.64–8.5)
Fibrinogen (g/L)	4.7 ± 1.13
ESR (mm/hr)	10 (5–22)

Values are presented as mean ± standard deviation or median (25th to 75th percentile values), as appropriate. BMI, body mass index; CR, chronotropic response; SBP, systolic blood pressure; DBP, diastolic blood pressure; HR, heart rate; VO_2PEAK_, volume of oxygen; RER, respiratory exchange ratio; HDL, high-density lipoprotein; LDL, low-density lipoprotein; WBC, white blood cells; HOMA, homeostasis model assessment insulin resistance; QUICKI; quantitative insulin sensitivity check index; hsCRP, high-sensitivity C-reactive protein; ESR, erythrocyte sedimentation rate.

**Table 2:** Correlation analysis between chronotropic responses with the study’s main outcome variables.

Variable		
Age	0.08	0.4
Gender	0.16	0.1
BMI	−0.26	0.01
Cardiorespiratory measurements		
Resting SBP	0.12	0.25
Resting DBP	0.15	0.14
VO_2PEAK_	0.34	<0.001
RER	0.33	0.001
Serological measurements		
LDL	0.17	0.09
HDL	0.38	<0.001
Triglycerides	0.02	0.84
WBC	−0.17	0.12
HOMA	−0.26	0.01
QUICKi	0.22	0.04
hsCRP	−0.2	0.05
Fibrinogen	−0.2	0.05
ESR	−0.16	0.12

BMI, body mass index; CR, chronotropic response; SBP, systolic blood pressure; DBP, diastolic blood pressure; VO_2peak_, peak volume of oxygen; RER, respiratory exchange ratio; LDL, low-density lipoprotein; HDL, high-density lipoprotein; WBC, white blood cells; HOMA, homeostasis model assessment insulin resistance; QUICKI; quantitative insulin sensitivity check index; hsCRP, high-sensitivity C-reactive protein; ESR, erythrocyte sedimentation rate.

**Table 3:** Linear regression model for the factors associated with chronotropic response.

Variable	β	t (p)
BMI	−0.2	−0.18 (0.9)
VO_2PEAK_	0.16	1.3 (0.2)
RER	0.33	3.7 (<0.001)
HDL	0.32	3.7 (0.001)
HOMA	0.22	1.12 (0.26)
QUICKi	0.26	1.14 (0.26)
hsCRP	−0.12	−1.01 (0.32)
Fibrinogen	0.04	0.28 (0.78)
R^2^ and *p* value of the model	0.37	<0.001

BMI, body mass index; VO_2peak_, peak volume of oxygen; RER, respiratory exchange ratio; HDL, high-density lipoprotein; HOMA, homeostasis model assessment insulin resistance; QUICKI; quantitative insulin sensitivity check index; hsCRP, high-sensitivity C-reactive protein.

## Data Availability

Research data supporting this publication are available upon request from the author.
